# The Whole-Genome Sequencing and Hybrid Assembly of *Mytilus coruscus*

**DOI:** 10.3389/fgene.2020.00440

**Published:** 2020-05-08

**Authors:** Ronghua Li, Weijia Zhang, Junkai Lu, Zhouyi Zhang, Changkao Mu, Weiwei Song, Herve Migaud, Chunlin Wang, Michaël Bekaert

**Affiliations:** ^1^Key Laboratory of Applied Marine Biotechnology, Ministry of Education, Ningbo University, Ningbo, China; ^2^Collaborative Innovation Center for Zhejiang Marine High-Efficiency and Healthy Aquaculture, Ningbo University, Ningbo, China; ^3^Faculty of Natural Sciences, Institute of Aquaculture, University of Stirling, Stirling, United Kingdom

**Keywords:** *Mytilus coruscus*, hard-shelled mussel, sequencing, genome assembly and annotation, mitochondria, syntheny

## Abstract

The hard-shelled mussel (*Mytilus coruscus*) is an economically important shellfish that has been cultivated for the last decade. Due to over-exploitation, most mussel stocks have dramatically declined. Efforts to study this species' natural distribution, genetics, breeding, and cultivation have been hindered by the lack of a high-quality reference genome. To address this, we produced a hybrid high-quality reference genome of *M. coruscus* using a long-read platform to assemble the genome and short-read, high-quality technology to accurately correct for sequence errors. The genome was assembled into 10,484 scaffolds, a total length of 1.90 Gb, and a scaffold N50 of 898 kb. *Ab initio* annotation of the *M. coruscus* genome assembly identified a total of 42,684 genes. This accurate reference genome of *M. coruscus* provides an essential resource with the advantage of enabling the genome-scale selective breeding of *M. coruscus*. More importantly, it will also help in deciphering the speciation and local adaptation of the *Mytilus* species.

## 1. Introduction

The marine mussel *Mytilus* is among the foremost cosmopolitan marine genera and is present in estuarine and oceanic habitats, in both the subtidal and intertidal zones (Koehn, 1991). The global distribution of *Mytilus* species combined with certain features, such as partial reproductive isolation, which produces natural hybrids in areas of sympatry (Hilbish et al., 2000), doubly uniparental inheritance (DUI) of mitochondria (Zouros et al., 1994), very high bio-accumulation, and a low bio-transformation potential for both organic and inorganic contaminants (Smolders et al., 2003), make them attractive models for genetic, evolution, and ecological research. Besides, mussels are commercially important molluscs: the global production of farmed mussels reached 2,164,000 tons in 2017 (Food and Agricultural Organization, 2019).

*Mytilus galloprovincialis* was the first sequenced marine mussel, and, its genome provided valuable information for the research and sustainable management of this species (Murgarella et al., 2016). *Mytilus coruscus* Gould, 1861 (also recognized as *Mytilus unguiculatus* Valenciennes, 1858) is another important mussel species distributed along the coast of China (Ye et al., 2012), Korea (An and Lee, 2012), and Japan (Okutani, 2000). In addition to its ecological importance in the intertidal and subtidal communities, it is also a popular edible shellfish in many Asian countries. As a large-bodied mussel species, *M. coruscus* is valued for its high nutritional value and good commercial price (Zhang et al., 2020). It has also been reported that some of its lipids have anti-inflammatory properties (Fu et al., 2015). The mariculture of *M. coruscus* has been carried out in several regions of China in the past; the Shengsi Islands (Zhejiang province) are one of the oldest and largest culture areas in the Eastern China Sea and have an annual production in excess of 500,000 tons (Guo et al., 2017). The cultured juveniles primarily originate from natural populations. In the last few decades, natural juvenile stocks have decreased, while mussel farms have increased (Shen et al., 2009). Recently, breeding programs of *M. coruscus* have been initiated, mainly aiming to improve growth rates and disease resistance under aquaculture conditions. Despite its ecological and economic importance, genome information on this endemic mussel species is still lacking.

This report presents the first draft genome assembly for *M. coruscus*, performed using a hybrid assembly strategy. An Oxford Nanopore Technologies PromethION long-read platform was used to assemble the genome and Illumina HiSeq X Ten short-read, high-quality technologies were used to accurately correct for sequence errors. The resulting assembled genome sequence has 10,484 scaffolds, a total length of 1.90 Gb, GC content of 32.22%, and a scaffold N50 of 898 kb. Furthermore, we identified 1.01 Gb (52.83% of the assembly) of repeat content, 42,684 protein-coding genes, 278 rRNAs, and a high heterozygosity of 1.64%. This high-quality reference genome will serve as a substantial resource for future studies of basic genetics as well as genome-scale selective breeding programs for *M. coruscus*.

## 2. Materials and Methods

### 2.1. Sample Collection and DNA Extraction

An adult female specimen of *M. coruscus* (133.73 g) was collected in November 2018 from the Shengsi Islands in Zhejiang province, China. Gills were dissected and stored in liquid nitrogen until DNA extraction. Genomic DNA was extracted as previously reported (Venier and Canova, 1996) with small modifications. Briefly, gill samples were digested with RNase and proteinase K, adjusted to 2% SDS, and heated at 60°C for 10 min. Sodium perchlorate was added, and DNA was extracted once by gently shaking with 24:1 chloroform:isoamyl alcohol for 30 min at room temperature. DNA was then precipitated with cold 75% ethanol and suspended in TE buffer (10 mM Tris–HCl, 1 mM EDTA, pH 7.4). The quality and concentration of the extracted genomic DNA were checked using 0.3% agarose gel electrophoresis and a Qubit fluorimeter (Invitrogen, Carlsbad, CA, USA).

### 2.2. Library Construction and Sequencing

High-quality DNA was used for subsequent library preparation and sequencing using PromethION and Illumina platforms (Biomarker Technologies Corporation, Beijing, China). To obtain long non-fragmented sequence reads, ~15 μg of genomic DNA was sheared and size-selected (30–80 kb) with a BluePippin (Sage Science, Beverly, MA, USA). The selected fragments were processed using the Ligation Sequencing 1D Kit (Oxford Nanopore, Oxford, UK) according to the manufacturer's instructions and sequenced using the PromethION DNA sequencer (Oxford Nanopore, Oxford, UK) for 48 h. For the estimation and correction of genome assembly, an Illumina DNA paired-end (PE) library with an insert size of 350 bp was constructed in accordance with the manufacturer's protocol and sequenced with an Illumina HiSeq X Ten platform (Illumina, Inc., San Diego, CA, USA) with paired-end 150 read layout.

### 2.3. *De novo* Assembly

**Figure 1:**
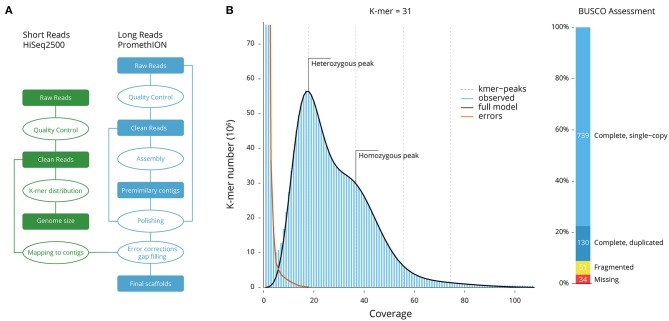
Genome size and quality estimations. **(A)** Genome assembly workflow. **(B)** The 31-mer distribution used for the estimation of genome size and heterozygosity. The heterozygous and homozygous peaks of k-mer depth are clearly markers, suggesting a high-complexity genome and high heterozygosity of 1.64%. **(C)** BUSCO assessment (Metazoa database; number of framework genes 954); 96.44% of the genes were recovered.

Reads from the two types of sequencing platforms were used at different assembly stages (Figure 1A). Long reads were filtered for length (>15,000 nt) and complexity (entropy over 15), while all short reads were filtered for quality (QC > 25), length (150 nt), absence of primers/adaptors, and complexity (entropy over 15) by using fastp (Chen et al., 2018). Using Jellyfish (Marçais and Kingsford, 2011), the frequency of 31-mers in the Illumina filtered data was calculated with a 1 bp sliding window (Vurture et al., 2017). Long reads were then assembled using wtdbg2 (Ruan and Li, 2020), which uses a fuzzy Bruijn graph. As it assembles raw reads without error correction and then builds the consensus from intermediate assembly output, multiple stages of error correction, gap closing, and polishing were applied. Original output was realigned against the long reads and polished using Minimap2 (Li, 2018) and Racon (Vaser et al., 2017), first with filtered reads to bridge potential gaps, and then with the filtered reads to correct for error. Finally, Pilon (Walker et al., 2014) was used to polish and correct for sequencing error using the short reads.

### 2.4. Gene Models

We used Braker (Hoff et al., 2019) to perform *ab initio* gene prediction, combining methods that integrate *ab initio* gene prediction and RNA-seq-based prediction to annotate the protein-coding genes in the *M. coruscus* genome. These raw RNA-seq reads were downloaded from the EBI for three independent transcriptomic projects, covering multiple tissues and multiple conditions: PRJNA301064 (Xu et al., 2016), PRJNA269003 & PRJNA269004. The resulting predictions were then filtered for the presence of at least one InterPro (Jones et al., 2014) pattern using InterProScan (Mitchell et al., 2019).

### 2.5. Repeat Sequences

The transposable elements were annotated using a *de novo* prediction by using RepeatModeler (Smit and Hubley, 2017) and LTR-Finder (Stanke et al., 2008). The repetitive sequences returned from these two algorithms were combined to compile a non-redundant repeat sequence library. With this library, we scanned the representative sequences in the *M. coruscus* genome using RepeatMasker (Smit et al., 2019).

### 2.6. Completeness

The completeness of gene regions was further assessed using BUSCO (Simão et al., 2015), using a Metazoa (release 10) benchmark of 954 conserved Metazoa genes.

### 2.7. Synteny With *M. galloprovincialis*

To assess the macro-synteny between *M. coruscus* and *M. galloprovincialis* (Murgarella et al., 2016) genomes, we reciprocally mapped all *M. coruscus* and *M. galloprovincialis* scaffolds with a minimum overlap of 100 nt.

### 2.8. Code Availability

The versions, settings, and parameters of the software used in this work are as follows:

Genome assembly: (1) **fastp**: version 0.20.0, short-read parameters: -q 25 -y -Y 15 -l 150 -detect_adapter_for_pe; (2) **fastp**: version 0.20.0, long-read parameters: -Q -y -Y 15 -l 15000; (3) **wtdbg2**: version 2.4, parameters: -x rs -k 23 -p 0 -AS 6 -R -g 1567m -rescue-low-cov-edges; (4) **wtpoa-cns**: version 2.4, default parameters; (5) **minimap2**: version 2.17, parameters: -x map-ont -r2k; (6) **racon**: version 1.4.3, default parameters; (7) **bwa**: version 0.7.17, mode mem, default parameters; (8) **pilon**: version 1.23, parameters: -diploid -fix all -changes; (9) **BUSCO**: version 4.0.2, parameters: -l metazoa_odb10; (10) **RepeatModeler**: version 1.0.11, parameters: -database mussel; (11) **LTR_Finder**: version 1.07, default parameters; (12) **RepteatMasker**: version 4.0.9, parameters: -lib mussel-families.fa; (13) **Braker**: version 2.1.4, parameters: -gff3 -softmasking; (14) **InterProScan**: version 5.42-78.0, parameters: -f tsv -dp -iprlookup -goterms.

K-mer analysis: (1) **jellyfish**: version 2.3.0, parameters: -m 31 -C -s 10G; (2) **GenomeScope**: version 2.0, default parameters.

Mitochondria annotation: (1) **MITOS**: revision 999, online version, parameters: “Genetic code 5”.

Synteny blocks: (1) **nucmer**: version 3.23, parameters: -simplify -maxgap = 500 -mincluster = 100; (2) **circos**: version 0.69-9, default parameters (tutorial 5.9).

## 3. Results and Discussion

### 3.1. Sequencing Results

After sequencing with the PromethION platform, a total of 11.31 million (161.04 Gb) long reads were generated, and these were used for the subsequent genome assembly. The N50 of the sequences produced was 21,771 nt. The Illumina HiSeq X Ten platform produces 288.22 million (86.47 Gb) paired-ended short reads (150 nt). The genome size of *M. coruscus* was estimated to have 2n = 28 chromosomes (Pérez-García et al., 2014) and a C-value of 1.90 pg (Ieyama et al., 1994) or 1.85 Gb; therefore, the average sequencing coverage was 87× and 46×, respectively (Table 1).

**Table 1:** Sequencing data statistics.

**Category**	**Number/length**
Total number of long reads	11,312,815
Total number of bases	161,041,744,749
N50 length	21,771 nt
Maximum read length	259,852 nt
Coverage	87×
Total number of PE short reads	288,220,402
Total number of bases	86,466,120,600
Read length	150 nt
Coverage	46×

### 3.2. *De novo* Assembly of the *M. coruscus* Genome

The frequency of 31-mers in the Illumina filtered data was calculated using Jellyfish and followed a Poisson distribution (Figure 1B). The proportion of heterozygosity in the *M. coruscus* genome was evaluated to be 1.64%, and the genome size was estimated as 1.57 Gb, with a repeat content of 36.35% (Table 2). Long-read assembly using wtdbg2 (Ruan and Li, 2020), polished using Racon and sequence-corrected using short reads and Pilon, produced an assembled genome of *M. coruscus* containing 10,484 contigs with a total length and contig N50 of 1.90 Gb and 898 kb, respectively (Table 2).

**Table 2:** Statistics of the genome assembly of *Mytilus coruscus*.

**Category**	**Number/length**
K-mer = 31	4,311,539,104
Heterozygous peak	18.87
Homozygous peak	37.74
Estimated genome size	1,567,289,679 nt
Estimated repeats	530,204,285 nt
Estimated heterozygosity	1.64%
Largest contig	11,437,774 nt
Total length	1,903,799,720 nt
N50	664,188 nt
Largest scaffolds	13,847,550 nt
Total length	1,903,825,920 nt
N50	898,347 nt
GC	32.22%
Mapped	98.42%
Properly paired	77.04%
Avg. coverage depth	138x
Coverage over 10×	99.48%
N's per 100 kbp	1.38
BUSCO recovered	96.44%
Predicted rRNA genes	278
Predicted gene models	92,615
Predicted protein-coding genes	42,684

### 3.3. Repeat Sequences and Gene Models

The transposable elements and repetitive sequences were annotated using RepeatMasker and LTR-Finder. In total, 1.01 Gb (52.83%) of the genome was identified as repetitive sequences (Table 3).

**Table 3:** RepeatMasker statistics.

**Element**	Number of elements^*^	**Length occupied (bp)**	**Percentage of sequence (%)**
SINEs	2,854	525,572	0.03
ALUs	0	0	0.00
MIRs	0	0	0.00
LINEs	437,682	160,984,195	8.46
LINE1	812	607,529	0.03
LINE2	13,314	5,148,240	0.27
L3/CR1	7,119	3,117,407	0.16
LTR elements	35,692	25,465,347	1.34
ERVL	0	0	0.00
ERVL-MaLRs	0	0	0.00
ERV classI	0	0	0.00
ERV classII	675	176,007	0.01
DNA elements	74,846	21,072,684	1.11
hAT-Charlie	0	0	0.00
TcMar-Tigger	0	0	0.00
Unclassified	3,215,437	784,518,335	41.21
Small RNA	0	0	0.00
Satellites	1,170	118,198	0.01
Simple repeats	307,099	12,840,131	0.67
Low complexity	56,444	2,732,946	0.14
Total repeats		1,005,864,117	52.83

* *Repeats fragmented by insertions or deletions have been counted as one element*.

We used a combined method that integrates *ab initio* gene prediction and RNA-seq-based prediction to annotate the protein-coding genes in the *M. coruscus* genome. In total, 42,684 distinct gene models were annotated.

### 3.4. Evaluating the Completeness of the Genome Assembly

To estimate the quality of the hybrid genome assembly, short reads were aligned to the consensus genome, and 98.42% did align overall, suggesting that our assembly results contained comprehensive genomic information.

The completeness of the gene models was also assessed using BUSCO (Simão et al., 2015) using a Metazoa (release 10) benchmark of 954 conserved Metazoa genes: 91.09% had complete gene coverage (including 13.63% duplicated ones), 5.35% were fragmented, and only 3.56% were missing (Figure 1C). This largely supports a high-quality *M. coruscus* genome assembly and gene models.

### 3.5. Synteny With *M. galloprovincialis*

**Figure 2:**
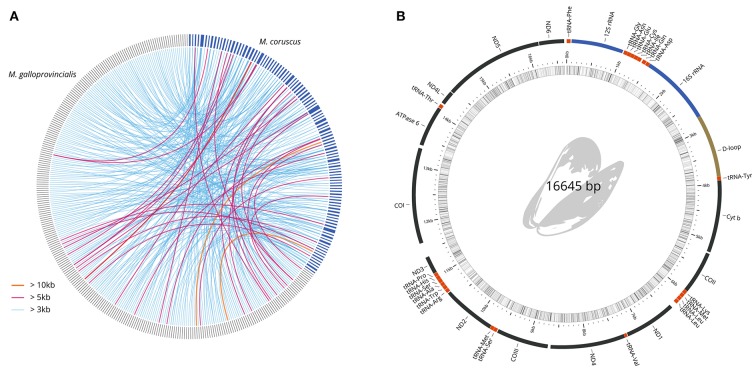
Genome comparisons. **(A)** Circos Krzywinski et al. (2009) mapping of longest synteny blocks between *M. galloprovincialis* (287 dark gray scaffolds) and *M. coruscus* (104 blue scaffolds). **(B)** *M. coruscus* annotated mitochondrial genome.

To uncover the macro-synteny between the *M. coruscus* and *M. galloprovincialis* (Murgarella et al., 2016) genomes, we reciprocally mapped all *M. coruscus* and *M. galloprovincialis* scaffolds with a minimum overlap of 100 nt (Table 4). A total of 28.12% of *M. galloprovincialis* scaffolds mapped to a *M. coruscus* scaffold while 70.25% *M. coruscus* scaffolds mapped to a *M. galloprovincialis* scaffold. Figure 2A reports the synteny blocks of over 3,000 nt; the map shows a good synteny between the two genomes despite *M. galloprovincialis* genome fragmentation.

**Table 4:** Comparison between *Mytilus* spp. assemblies.

**Category**	M. galloprovincialis^*^	***M. coruscus***
Num. scaffolds	1,002,334	10,484
Span	1,500,149,602 nt	1,903,825,920 nt
Longest scaffold	67,529 nt	13,847,550 nt
Shortest scaffold	200 nt	3,201 nt
N50	2,931 nt	898,347 nt
GC	31.71%	32.22%
Syntenic	281,841 (28.12%)	7,365 (70.25%)

* *Sequences deposited and reported by Murgarella et al. (2016) differ, as only sequences over 200 nt were publicly deposited*.

### 3.6. Mitochondrial Genome

The mitochondrial genome was manually recovered from the genome assembly. The contig was validated for continuity and circularity and annotated using MITOS (Bernt et al., 2013). The complete mitochondrial genome (Figure 2B) was compared to the reference *M. coruscus* genome (Lee and Lee, 2016). Only one haplotype was recovered, which differed from the reference by only 6 SNPs and one 2-nucleotide insertion.

## 4. Conclusion

This study is the first to present a high-quality genome sequence assembly of the hard-shelled mussel *M. coruscus*. We generated a hybrid genome assembly of 1.90 Gb with an N50 of 898 kb. The assembled genome was predicted to contain 42,684 protein-coding genes, 278 rRNAs, 1.01 Gb (52.83%) of repetitive elements, and high heterozygosity of 1.64%. We also recovered and annotated the whole circular mitochondrial genome of 16.65 kb lacking *atp8* gene. This high-quality reference genome will serve as a substantial resource for future studies of basic genetics as well as genome-scale selective breeding programs for *M. coruscus*.

## Data Availability Statement

The raw sequencing reads of all libraries are available from EBI/ENA via the accession numbers ERR3415816 and ERR3431204. The assembled genomes are available in EBI with the accession numbers ERZ1292486 (nuclear genome) and ERZ1195933 (mitochondrial genome), Project PRJEB33342.

## Ethics Statement

The animal study was reviewed and approved by Animal Care and Use committee at the School of Marine Sciences, Ningbo University.

## Author Contributions

RL, MB, and CW conceived, initialized, and guided the entire project. JL, ZZ, CM, and WS collected the sample. WZ, JL, and ZZ prepared the sample and performed the genome sequencing. MB performed the data processing and genome, and gene model analysis. RL and MB drafted the manuscript. HM helped with the manuscript preparation. All authors read and approved the final manuscript.

## Conflict of Interest

The authors declare that the research was conducted in the absence of any commercial or financial relationships that could be construed as a potential conflict of interest.
